# Evaluating Metacognitive Therapy to Improve Treatment of Anxiety and Depression in Cardiovascular Disease: The NIHR Funded PATHWAY Research Programme

**DOI:** 10.3389/fpsyt.2022.886407

**Published:** 2022-06-03

**Authors:** Adrian Wells, David Reeves, Calvin Heal, Linda M. Davies, Gemma E. Shields, Anthony Heagerty, Peter Fisher, Patrick Doherty, Lora Capobianco

**Affiliations:** ^1^Division of Psychology and Mental Health, Faculty of Biology, Medicine and Health, School of Health Sciences, Rawnsley Building, Manchester Royal Infirmary, The University of Manchester, Manchester, United Kingdom; ^2^Greater Manchester Mental Health NHS Foundation Trust, Rawnsley Building, Manchester Royal Infirmary, Manchester, United Kingdom; ^3^NIHR School for Primary Care Research, Williamson Building, Manchester Academic Health Science Centre, The University of Manchester, Manchester, United Kingdom; ^4^Jean McFarlane Building, Faculty of Biology Medicine and Health, Centre for Biostatistics, School of Health Sciences, The University of Manchester, Manchester, United Kingdom; ^5^Division of Population Health, Health Services Research and Primary Care, Jean McFarlane Building, Faculty of Biology Medicine and Health, Centre for Health Economics, School of Health Sciences, The University of Manchester, Manchester, United Kingdom; ^6^Core Technology Facility, The University of Manchester School of Medical Sciences, Manchester, United Kingdom; ^7^Manchester University NHS Foundation Trust, Manchester Royal Infirmary, Manchester, United Kingdom; ^8^Waterhouse Building, Block B, Institute of Psychology, Health and Society, University of Liverpool, Liverpool, United Kingdom; ^9^The Royal Liverpool and Broadgreen University Hospital NHS Trust, Liverpool, United Kingdom; ^10^Department of Health Sciences, Seebohm Rowntree Building, University of York, York, United Kingdom

**Keywords:** metacognitive therapy, anxiety, depression, cardiac rehabilitation, qualitative, health economic, randomized control trial

## Abstract

**Background:**

Anxiety and depression contribute to poorer physical and mental health outcomes in cardiac patients. Psychological treatments are not routinely offered in cardiac care and have mixed and small effects. We conducted a series of studies under the PATHWAY research programme aimed at understanding and improving mental health outcomes for patients undergoing cardiac rehabilitation (CR) through provision of metacognitive therapy (MCT).

**Methods:**

PATHWAY was a series of feasibility trials, single-blind, multicenter, randomized controlled trials (RCTs), qualitative, stated preferences for therapy and health economics studies.

**Findings:**

Patients felt their psychological needs were not met in CR and their narratives of distress could be parsimoniously explained by the metacognitive model. Patients reported they would prefer therapy over no therapy as part of CR, which included delivery by a cardiac professional. Two feasibility studies demonstrated that RCTs of group-based and self-help MCT were acceptable, could be embedded in CR services, and that RCTs of these interventions were feasible. A definitive RCT of group-MCT within CR (*n* = 332) demonstrated significantly greater reductions in the severity of anxiety and depression, exceeding CR alone, with gains maintained at 12 month follow-up (SMD HADS total score = 0.52 at 4 months and 0.33 at 12 months). A definitive trial of self-help MCT is ongoing.

**Conclusion:**

There is a need to better meet the psychological needs of CR patients. Embedding MCT into CR demonstrated high acceptability and improved efficacy on psychological outcomes. Results support roll-out of MCT in CR with evaluation of national implementation.

**Registration:**

URL: NCT02420431; ISRCTN74643496; NCT03129282.

## Introduction

Cardiovascular disease (CVD) is the most common non-communicable disease and makes the largest contribution to morbidity and mortality worldwide (1). The psychological impacts of CVD are substantial and recognized as contributing to poorer prognosis (2). Specifically, anxiety and depression, which affect up to one-third of people with CVD, have been linked to increased future cardiac events, poorer quality of life, greater healthcare costs and poorer long-term psychological adjustment (3). Effective management of anxiety and depression in coronary care is crucial and a priority for policy makers and future clinical trials (4). Unfortunately, the quality of evidence supporting psychological treatments for anxiety and depression in CVD is generally low with small sample sizes impacting reliability of findings (5). The treatment effects have been found to be small to moderate (5).

Cardiac rehabilitation (CR) is offered routinely in a group setting to patients following a cardiac event, it reduces mortality and hospital readmissions, and improves quality of life (6, 7). CR consists of exercise sessions, education and stress management techniques directed at improving CVD risk profiles, physical fitness and psychological functioning (6). The psychological components are not standardized and vary by CR programme but may include counseling, relaxation, meditation, and cognitive challenging of negative thoughts. However, systematic reviews and meta-analyses suggest that the effects on anxiety and depression symptoms are small to moderate, and the quality of evidence is low suggesting major uncertainty over the effects reported. It is recommended that further studies be conducted to test the effectiveness of psychological therapies (5).

Metacognitive therapy (MCT) (8, 9) is a recent manualized treatment approach that may be particularly suited to addressing the psychological needs of CR patients. This is because MCT, unlike other therapies, does not require an in-depth analysis and challenging of the content of patients worries (e.g., “what if I suffer another heart attack”) that in the CR context are often realistic (10). In contrast, MCT focuses on regulating repetitive negative thinking cycles such as worry and rumination and other unhelpful behaviors that research shows maintain anxiety and depression.

MCT is based on a model where anxiety and depression are maintained by common factors involving difficult to control repetitive negative thinking and attention to threat. Feeding this thinking pattern are distorted beliefs (metacognitive beliefs) about thoughts concerning the usefulness of worrying (e.g., “Thinking the worst about symptoms will keep me safe”) and beliefs concerning the uncontrollability and danger of thinking (e.g., “I have lost control of worrying”). These beliefs interfere with the effective regulation of repetitive negative thinking patterns that maintain distress. For example, in response to a negative thought such as “what if I have another heart attack” the individual who believes that interpreting symptoms in the worst way possible will keep them safe engages in sustained negative thinking. Similarly, beliefs that worry cannot be controlled leads to diminished effort (or unhelpful strategies; e.g., alcohol) in controlling repetitive negative thinking. Such thinking persists leading to an escalating sense of threat and corresponding feelings of anxiety and sadness. MCT helps the person to consciously identify and change this thinking pattern by bringing worry, rumination and an excessive tendency to focus on threat (e.g., chest sensations) under control. A key therapeutic process in doing so is the challenging of metacognitive beliefs behind this thinking pattern. Results from randomized controlled trials in mental health settings and meta-analyses demonstrate that MCT is highly efficient and effective for anxiety and depression and may be more effective than various forms of cognitive behavior therapy (11). An important question concerns whether such positive effects might translate to the context of treating mental health symptoms in CR patients.

Given the limitations of existing treatments, there is a priority to implement effective psychological treatments in CR. Such a need led to the PATHWAY research programme, funded by the UK NIHR to examine the needs of patients and the effects of MCT. The PATHWAY program set out to address an important question; Can we improve psychological outcomes of CR patients? In doing so we explored the needs of patients, the feasibility of conducting a trial in the CR context and the effectiveness of adding MCT to usual CR. In the remainder of this paper, we summarize the results of the PATHWAY programme, which included the first large-scale trial of the effectiveness of adding Metacognitive Therapy for anxiety and depression in cardiovascular disease (CVD) patients.

## Identifying Psychological Needs of CR Patients

Few studies have evaluated why the effects of psychological treatments are so limited in CVD. We aimed to explore: (1) how patients described their psychological distress, (2) if CR and routine care addressed their psychological needs, and (3) how two different psychological treatment models might conceptualize patient problems.

To assess how patients described their psychological distress, we conducted semi-structured qualitative interviews with 46 CR patients with elevated symptoms of anxiety and/or depression. Data were analyzed using a constant comparative approach (10). Following the first four interviews commonalities and contrasts in patients accounts were noted and used to develop a preliminary thematic framework based on the research questions. Data continued to be analyzed into categories that described emerging elements of patients' accounts (10). Patients described low mood and diverse concerns and anxieties, not limited to fear of another cardiac event. Patients described worrying constantly, worrying about their worry, and feeling that worry was uncontrollable and harmful. Patients wanted to “get back to normal” but lacked any sense of how to achieve this. In fact, they reported that they were reluctant to discuss their worries with CR staff. They hoped to recover over time, meanwhile seeking reassurance that they were responding “normally.” Patients were mostly dismissive of psychological techniques used in CR. We interpreted these results as suggesting that an acceptable psychological intervention for patients might be one that: (1) reduces a range of concerns that extend beyond a patient's cardiac event, (2) challenges beliefs about the uncontrollability and harmfulness of worry, (3) offers skills they could practice, (4) allows them to keep the content of worries private if they choose to.

These data appeared to support the choice of a transdiagnostic treatment such as MCT that targets the regulation of worry. Furthermore, patients' worry could be addressed with MCT without focussing on the content of those worries which seemed to be more consistent with the views expressed. The identification of patient concerns about needing to know if they were responding “normally” and their “worry about worry” were consistent with an MCT interpretation, which emphasizes developing and modifying unhelpful beliefs about thinking.

Subsequently, we explored whether the distress reported by 49 CR patients could be conceptualized parsimoniously within a MCT model of causal mechanisms (12) in contrast to the CBT model. Analysis of transcripts followed a stepwise approach. First inductive analysis followed a constant comparative approach whereby we identified commonalities and contrasts in how patients described and understood their distress. Patient transcripts were then re-examined from the perspectives of the CBT and MCT models using deductive and inductive elements. Inductive analysis allowed us to assess which model had the best fit with patients experience, with the primary criterion for fit being parsimony (i.e., how simply the models could be related to patients' accounts of their distress). Four patient transcripts reflecting diversity in the presentation and clinical context of distress were then selected for detailed analysis to illustrate the divergence between CBT and MCT models. When analyzing from the perspective of the CBT model, we focused on identifying distinct negative automatic thoughts, and then categorized the cognitive distortions apparent in these (13). When analyzing from the perspective of the MCT model, we focused on identifying talk showing perseverative negative thinking (i.e., worry or rumination). When we saw possible underlying beliefs—negative beliefs from the perspective of CBT, or metacognitive beliefs from the perspective of MCT—these were coded and re-examined from the alternative perspective.

Patient talk showed multiple types of cognitive distortions, and required a distinction between realistic and unrealistic thoughts which proved difficult in the context of risk of future cardiac events. Understanding emotional distress from a CBT perspective presented multiple, diverse targets for treatment. In contrast, from the perspective of MCT, a single category of *perseverative negative thinking* was sufficient to understand all talk, irrespective of whether it contained realistic or unrealistic thoughts. MCT appeared to provide a more parsimonious account of emotional distress.

The data gained from our qualitative analyses supported the choice of MCT as a potential treatment approach for meeting the psychological needs of CR patients. However, we did not know if MCT was an acceptable treatment to patients or feasible to evaluate within CR.

## A Feasibility Trial of Group-MCT (NCT02420431; ISRCTN74643496)

We assessed the acceptability and feasibility of implementing a randomized controlled trial of group MCT within CR services to support a subsequent definitive trial (14). We used the data from the study to estimate the required sample size, recruitment rates and site numbers, and should no modification to methods be required intended to use participants' data as an internal pilot in a subsequent full trial if deemed appropriate.

The evaluation of MCT was based on the implementation of a group-based treatment manual (15). The treatment consisted of six-sessions to explore and modify flexibility and control over patterns of extended negative thinking and to modify unhelpful metacognitive beliefs. At the end of treatment, each patient received their own “helpful behaviors” prescription summarizing what they had learned. Homework practice of certain techniques and behaviors was a feature throughout.

CR included 8 to 10 weeks of group exercise and educational sessions lasting 45 to 60 min. Exercise seminars focused on a range of topics including lifestyle and medical risk factor management. All sites included a psychological component within CR, however the content of these components varied. All sites delivered sessions on relaxation including breathing techniques and progressive muscle relaxation, however some sites provided further psychological components. For example, two sites incorporated cognitive therapy methods (i.e., challenging negative thoughts, worry decision tree), while one site delivered psychoeducational information on stress. In addition, one site offered a 4 week stress management course as part of CR which included generating and sharing a cognitive-behavioral case formulation, mindfulness techniques, and individual counseling with an occupational therapist.

Fifty-two patients with elevated anxiety and/or depression (Hospital Anxiety and Depression Scale (HADS) (16) > 8 on at least one subscale) were randomly allocated to group-MCT plus usual CR (MCT+CR; *n* = 23) or usual CR alone (*n* = 29). Three NHS hospital sites participated across the North West of England. For further details on the study protocol see Wells et al. (17). Analysis of primary and secondary symptom outcomes was purely descriptive and based on the whole sample (not individual arms) as feasibility trials are not powered to detect differences and it was necessary to maintain the blinding of the research team if the data was to be combined with that of a larger subsequent trial. The assessment of feasibility and acceptability of adding group-MCT to CR was based on recruitment rates, withdrawal, and drop-out by the primary end-point of 4 months; number of MCT and CR sessions attended; completion of follow-up questionnaires; and ability of the outcome measures to discriminate between patients. The study was also used to re-estimate the required sample size for a full-scale trial. We also examined the extent by which non-mental health specialists (cardiac staff) adhered to the Group-MCT protocol.

Patient attendance at CR was high with 75% of participants across both arms attending usual CR, which is in line with national CR data (18). Group-MCT did not impact on attendance at usual care, as CR completion rates did not differ by study arm. In addition, attendance at group-MCT was high, with over half of patients attending at least four (the minimum pre-defined dose) out of six group-MCT sessions. At 4 month follow-up 72.4 and 69.6% of control and intervention group participants, respectively, returned follow up questionnaires. All outcome measures demonstrated a good range of observed scores, with little in the way of floor or ceiling effects.

We examined therapists' level of adherence to the treatment protocol since the treatment was to be administered by non-mental health specialists with no experience of delivering psychological treatments. An adherence checklist was used, where therapists indicated which aspects of the manual they had and had not implemented in each session. Checklist items were based on the key aspects of the manual that were to be implemented in each session. For example, in the first session adherence items included completing the case formulation, socializing patients to the model, practicing an attention control exercise called SpACE, and assigning homework. A total adherence score was created for each session by summing the total number of elements completed in session. Adherence was high across all three sites, with an overall adherence rating of 98.2%.

We estimated that a definitive randomized controlled trial would require a recruitment sample of 332 to have 90% power to detect a target 0.4 effect size (Cohen's d). This estimate took into account results from the internal pilot which indicated an attrition rate of 35%, over-time correlation of 0.5 (unchanged), mean group size of 3 and ICC of 0.05 (assumed).

## Assessment of Patient Views of MCT

Alongside the feasibility (and full-scale trial), we conducted a qualitative study of CR patients views of participating in group MCT (19). In-depth qualitative interviews with 24 purposively sampled CR patients following group MCT were conducted. Data was analyzed using thematic analysis and revealed two main themes: (1) general therapy factors that were seen largely as beneficial, where patients highlighted interaction with other CR patients and CR staff delivery of treatment and their knowledge of cardiology; (2) MCT-specific factors that were seen as beneficial which included particular treatment techniques. All the patients who completed group MCT were positive about it and described self-perceived changes in their thinking and well-being. A minority of patients (*n* = 4) that were interviewed did not complete the intervention and two of them gave specific reasons for not finding the treatment helpful. They described how the staff delivering the intervention referred repeatedly to the treatment manual and this gave the impression that there was a lack of knowledge about the intervention being delivered.

## Full-Scale Trial of Effectiveness of group-MCT in CR (NCT02420431; ISRCTN74643496)

As group-MCT appeared to be feasible and acceptable to deliver within CR, the next step was to evaluate the effectiveness of group-MCT plus CR in comparison to usual CR alone in a fully powered RCT (20).

The feasibility trial determined that a full trial required no changes to trial procedures, including recruitment and randomization processes, outcome measures, or the MCT manual or treatment delivery. We therefore regarded the 52 patients from the feasibility trial as a valid internal pilot to the full trial and recruited a further 280 CR patients –giving a total sample of 332—from five NHS hospitals across the North-West of England.

In total, 163 patients (49.1%) were randomly allocated to MCT+CR and 169 (50.9%) to usual CR alone. The primary outcome was the Hospital Anxiety and Depression Scale (HADS) total score after treatment (4 month follow-up). Secondary outcomes were the HADS anxiety and depression sub-scale scores, traumatic stress symptoms, and psychological mechanisms including metacognitive beliefs and repetitive negative thinking. Analysis was intention to treat. The adjusted group difference on the primary outcome, HADS total score at 4 months, significantly favored the MCT+CR arm (−3.24 [95% CI, −4.67 to −1.81], *p* < 0.001, standardized effect size 0.52 (95% CI 0.291 to 0.75). The difference was smaller but remained significant at 12 months (−2.29 [95% CI −3.72 to −0.66] *p* < 0.01; standardized effect size 0.33 [95% CI 0.101 to 0.568]). The MCT intervention improved outcomes significantly for both anxiety and depression HADS subscales when assessed separately at 4 months and for anxiety but not depression at 12 months. Most secondary outcomes also favored MCT with medium to large effects observed and maintained up to 12 months. An incidental finding (though not statistically significant) was that the addition of MCT appeared to lower the risk of psychological deterioration during CR. No adverse treatment-related effects were reported. An economic evaluation is planned and will be published separately (21).

As part of the PATHWAY study a systematic review was conducted to evaluate the current cost-effectiveness literature for CR (7). The review identified that CR is cost-effective, however very little evidence focuses on psychological therapy in CR and this evidence has more mixed results. It found that more research is needed to determine the most cost-effective design of CR (inclusive of psychological intervention).

## Improving Access: a Feasibility and Acceptability Trial of Home-MCT (NCT03129282)

While CR offers home-based options, this has not been extended to psychological support which is typically offered in face-to-face formats (22). We therefore conducted a feasibility trial to evaluate the feasibility and acceptability of a home-based (i.e., self-help) version of the metacognitive therapy (23, 24). As with the feasibility trial of group-MCT, this trial was designed to also support a subsequent definitive trial (NCT03999359) (24) should the findings be favorable.

A multicenter randomized controlled trial with 4- and 12 month follow-up comparing self-help MCT plus usual CR (intervention) vs. usual CR alone (control) was conducted in two NHS hospitals in the North West of England. One-hundred and eight participants (69 males, 39 females) participated in a randomized, single-blind, parallel trial. Patients had a mean age of 59.9 years (SD = 9.7, range: 40–84).

Retention in both the intervention and control groups was high, with 52 (96.3%) patients in the control arm and 45 (83.3%) patients in the intervention arm returning questionnaires at 4 month follow up. This was maintained at 12 month follow up with 90.7% of control and 81.5% of intervention participants returning follow up data. All outcome measures demonstrated a wide range of observed scores, covering most of the possible range, with little in the way of floor or ceiling effects. Engagement with Home-MCT was high, 72.7% of patients who returned the end of study questionnaire completed four or more of the six modules. While most patients reported completing a module in 60 min, individual times varied, ranging from 40 to 105 min. Home-MCT demonstrated high credibility, with patients stating they found the manual easy to use and understand (median rating of 80 out of 100), that the homework was easy to follow (median rating of 85 out of 100), and that the exercise SpACE was easy to use (median rating of 90 out of 100).

Overall, Home-MCT was found to be an acceptable and feasible addition to CR. Home-MCT demonstrated no reported adverse events. The results support progression to a full scale randomized controlled trial to evaluate the efficacy of Home-MCT.

## Exploring Patient Preferences For Treatment

Discrete choice experiments (DCE) are used to understand preferences for healthcare interventions and services (25, 26), whereby participants are asked to choose between hypothetical scenarios that vary by key attributes. A DCE was used to explore the preferences of participants who participated in the Home-MCT feasibility study for attributes of a psychological therapy intervention, relevant to home-based care. The aim was to evaluate the feasibility of conducting a full DCE, estimate the sample size needed for a full study and to explore preliminary preferences for included attributes.

Patient and public involvement feedback was sought to aid in selecting and defining attribute and levels (27). Thirty-five participants took part in the DCE. Participants stated that they disliked having no information about therapy before it started and favored lower costs to the NHS (28). Participants appeared to favor home-based therapy, with reduced waiting times and online or smartphone assisted therapy, though these results were not statistically significant. Significant positive constants for therapy options suggest that participants highly valued receiving therapy (compared to no therapy). It was estimated that a sample size of around 370 would be needed to identify significant coefficients for most attributes. The pilot study demonstrated the feasibility of a DCE in this group, it identified potential attributes and levels, and estimates the sample sizes needed for a full study.

## General Discussion

Current psychological support offered to CVD patients within cardiac services is limited in scope and in efficacy. It is imperative that new and more effective psychological therapies are offered that can be embedded within services.

In addressing this issue, we conducted a systematic research programme exploring the psychological needs of CR patients (10), the goodness of fit of metacognitive therapy to patient problems (12), the feasibility of MCT (14), the effectiveness of MCT (20), preferences for treatment attributes (28) and the feasibility and acceptability of a home-based self-help MCT manual and trial (23). We also examined preliminary health-economics data for the intervention.

Patients within CR feel that their psychological needs are not being effectively met by the components of CR, they have multiple concerns and are reluctant to talk about them in the CR context (10). The wide-ranging personal concerns expressed could be explained by a single construct; perseverative negative thinking, the reduction of which is a key target of MCT (12). The feasibility and acceptability of a RCT of MCT was supported by a feasibility trial (14).

A definitive RCT demonstrated that adding group-MCT to CR significantly reduced anxiety and depression and conferred advantages on a range of secondary psychological outcomes, with most gains sustained over 12 months (20). These results compared favorably against previous studies of psychological therapies (5). It was also shown that running group-MCT alongside usual CR did not interfere with CR attendance and engagement. We did not set out to test whether MCT is more effective than another specific psychological treatment but aimed to answer a more pragmatic question that could have immediate impact; can the addition of MCT to usual CR improve psychological outcomes? It is possible that a different model of treatment (or simply more therapist contact) might also produce improved outcomes. Future studies are required that might assess the relative effectiveness of MCT vs. other therapeutic approaches when combined with CR. Results of studies from mental health settings are increasingly suggesting that MCT might outperform other evidence-based approaches (11, 29).

A test of a self-help version of MCT when added to CR was found to be a feasible and acceptable trial to pursue, potentially increasing patient choice (23). An investigation of patient preferences for therapy attributes showed that patients favored being offered a psychological therapy rather than no therapy at all, that they would prefer information about treatment before it was offered, and that they would prefer a home-based treatment option (28).

The results of the PATHWAY programme present implications for clinical practice and the development of more psychologically-oriented CR approaches. In particular, the results highlight the improvement in outcomes that could be achieved by using clinic-based group-metacognitive therapy. However, a home-based approach could offer more choice and is acceptable, but evidence of the effectiveness of self-help MCT needs to be determined by a full-scale RCT, a study which is currently in progress (NCT03999359). In order to realize the service-level potential of MCT, future studies should examine implementation within healthcare services and cost-effectiveness.

## Author Contributions

AW and LC contributed to the first and subsequent drafts of the manuscript. All authors edited the manuscript and read and approved the final version.

## Funding

This paper presents independent research funded by the National Institute for Health Research (NIHR) under its Programme Grants for Applied Research (PGfAR) Programme (Grant Reference Number RP-PG-1211-20011).

## Author Disclaimer

The views expressed are those of the author(s) and not necessarily those of the NIHR or the Department of Health.

## Conflict of Interest

AW is the director of the MCT-Institute and developer of MCT. AW has also written books on CBT and MCT. The remaining authors declare that the research was conducted in the absence of any commercial or financial relationships that could be construed as a potential conflict of interest.

## Publisher's Note

All claims expressed in this article are solely those of the authors and do not necessarily represent those of their affiliated organizations, or those of the publisher, the editors and the reviewers. Any product that may be evaluated in this article, or claim that may be made by its manufacturer, is not guaranteed or endorsed by the publisher.

## References

[1] World Health Organization (WHO). WHO Global NCD Action Plan 2013–2020. WHO (2013). Available online at: https://www.who.int/nmh/events/ncd_action_plan/en/ (accessed June 10, 2020).

[2] Meijer A Conradi HJ Bos EH Thombs BD van Melle JP de Jonge P. Prognostic association of depression following myocardial infarction with mortality and cardiovascular events: a meta-analysis of 25 years of research. Gen Hosp Psychiatry. (2011) 33:203–16. 10.1016/j.genhosppsych.2011.02.007 21601716

[3] Palacios J Khondoker M Mann A Tylee A Hotopf M. Depression and anxiety symptom trajectories in coronary heart disease: associations with measures of disability and impact on 3-year health care costs. J Psychosom Res. (2018) 105:1–8. 10.1016/j.jpsychores.2017.10.015 29275777

[4] Department of Health. Cardiac Rehabilitation Commissioning Pack. London: Department of Health (2010).

[5] Richards SH Anderson L Jenkinson CE Whalley B Rees K Davies P. Psychological interventions for coronary heart disease. Cochrane Database Syst Rev. (2017) 4:CD002902. 10.1002/14651858.CD002902.pub4 28452408 PMC6478177

[6] Anderson L Oldridge N Thompson DR Oldridge N Zwisler AD Rees K. Exercise-based cardiac rehabilitation for coronary heart disease. Cochrane Database Syst Rev. (2016) 67:1–12. 10.1016/j.jacc.2015.10.044 26730878 PMC6491180

[7] Shields G Wells A Doherty P Heagerty A Buck D Davies L. Cost-effectiveness of cardiac rehabilitation: a systematic review. Heart. (2018) 104:1403–10. 10.1136/heartjnl-2017-312809 29654096 PMC6109236

[8] Wells A. Metacognitive Therapy for Anxiety and Depression. New York, NY; Guilford Press (2009).

[9] Wells A. Breaking the cybernetic code: understanding and treating the human metacognitive control system to enhance mental health. Front Psychol. (2019) 10:2621. 10.3389/fpsyg.2019.02621 31920769 PMC6920120

[10] McPhillips R Salmon P Wells A Fisher P. Cardiac rehabilitation patients' accounts of their emotional distress and psychological needs: a qualitative study. J Am Heart Assoc. (2019) 8:e011117. 10.1161/JAHA.118.011117 31433708 PMC6585358

[11] Normann N Morina N. The efficacy of metacognitive therapy: a systematic review and meta-analysis. Front Psychol. (2018) 9:2211. 10.3389/fpsyg.2018.02211 30487770 PMC6246690

[12] McPhillips R Salmon P Wells A Fisher P. Qualitative analysis of emotional distress in cardiac patients from the perspectives of cognitive behavioral and metacognitive theories: why might cognitive behavioral therapy have limited benefit, and might metacognitive therapy be more effective?. Front Psychol. (2019) 9:2288. 10.3389/fpsyg.2018.02288 30662413 PMC6328488

[13] Beck AT. Cognitive Therapy and the Emotional Disorders. New York, NY: International Universities Press (1976).

[14] Wells A Reeves D Heal C Fisher P Davies L Heagerty A. Establishing the feasibility of group metacognitive therapy for anxiety and depression in cardiac rehabilitation: a single bind randomized pilot study. Front Psychiatry. (2020) 11:582. 10.3389/fpsyt.2020.00582 32714216 PMC7344162

[15] Wells A. MCT-PATHWAY: Group Metacognitive Therapy Treatment Manual. Manchester: University of Manchester (2015).

[16] Zigmond AS Snaith RP. The hospital anxiety and depression scale. Acta Psychiatr Scand. (1983) 67:361–70. 10.1111/j.1600-0447.1983.tb09716.x 6880820

[17] Wells A McNicol K Reeves D Salmon P Davies L Heagerty A. Improving the effectiveness of psychological interventions for depression and anxiety in the cardiac rehabilitation pathway using group-based metacognitive therapy (PATHWAY Group MCT): study protocol for a randomised controlled trial. Trials. (2018) 19:215. 10.1186/s13063-018-2593-8 29615092 PMC5883514

[18] National Audit of Cardiac Rehabilitation (NACR). Annual Report. York: British Heart Foundation, University of York (2018). Available online at: https://www.bhf.org.uk/informationsupport/publications/statistics/national-audit-of-cardiac-rehabilitation-quality-and-outcomes-report-2018.

[19] McPhillips R Capobianco L Cooper B Husain Z Wells A. Cardiac rehabilitation patients experiences and understanding of group metacognitive therapy: a qualitative study. Open Heart. (2021) 8:e001708. 10.1136/openhrt-2021-001708 34261779 PMC8281095

[20] Wells A Reeves D Capobianco L Heal C Davies L Heagerty A. Improving the effectiveness of psychological interventions for depression and anxiety in cardiac rehabilitation: PATHWAY—a single-blind, parallel, randomized, controlled trial of group metacognitive therapy. Circulation. (2021) 144:23–33. 10.1161/CIRCULATIONAHA.120.052428 34148379 PMC8247550

[21] Shields GE Wells A Doherty P Reeves D Capobianco L Heagerty A. Protocol for the economic evaluation of metacognitive therapy for cardiac rehabilitation participants with symptoms of anxiety and/or depression. BMJ Open. (2020) 10:e035552. 10.1136/bmjopen-2019-035552 32912974 PMC7485258

[22] Whalley B Thompson D Taylor R. Psychological interventions for coronary heart disease: cochrane systematic review and meta-analysis. Int J Behav Med. (2012) 21:109–21. 10.1007/s12529-012-9282-x 23179678

[23] Wells A Reeves D Heal C Fisher P Davies L Heagerty A. Metacognitive therapy self-help for anxiety-depression: single-blind randomized feasibility trial in cardiovascular disease. Health Psychol. (2022) 41:366-77. 10.1037/hea0001168 35467904 PMC9037049

[24] Wells A McNicol K Reeves D Salmon P Davies L Heagerty A. Metacognitive therapy home-based self-help for cardiac rehabilitation patients experiencing anxiety and depressive symptoms: study protocol for a feasibility randomised controlled trial (PATHWAY Home-MCT). Trials. (2018) 19:444. 10.1186/s13063-018-2826-x 30115112 PMC6097432

[25] de Bekker-Grob EW Ryan M Gerard K. Discrete choice experiments in health economics: a review of the literature. Health Econ. (2012) 21:145–72. 10.1002/hec.1697 22223558

[26] Soekhai V de Bekker-Grob EW Ellis AR. Discrete choice experiments in health economics: past, present and future. Pharmacoeconomics. (2019) 37:201–26. 10.1007/s40273-018-0734-2 30392040 PMC6386055

[27] Shields GE Brown L Wells A Capobianco L Vass C. Utilising patient and public involvement in stated preference research in health: learning from the existing literature and a case study. Patient. (2021) 14:399–412. 10.1007/s40271-020-00439-2 32748242 PMC8205869

[28] Shields GE Wright S Wells A Doherty P Capobianco L Mary L. Delivery preferences for psychological intervention in cardiac rehabilitation: a pilot discrete choice experiment. Open Heart. (2021) 8:e001747. 10.1136/openhrt-2021-001747 34426529 PMC8383873

[29] Callesen P Reeves D Heal C Wells A. Metacognitive therapy versus cognitive behaviour therapy in adults with major depression: a parallel single-blind randomised controlled trial. Sci Rep. (2020) 10:7878. 10.1038/s41598-020-64577-1 32398710 PMC7217821

